# Utilizing ECG data in educational psychology research: insights and limitations

**DOI:** 10.3389/fpsyg.2025.1440721

**Published:** 2025-05-09

**Authors:** Sigrid Hackl-Wimmer, Helmut Karl Lackner, Marina Tanja Waltraud Eglmaier, Lars Eichen, Manuela Paechter

**Affiliations:** ^1^Department of Educational Psychology, Institute of Psychology, University of Graz, Graz, Austria; ^2^Division of Physiology, Otto Loewi Research Center, Medical University of Graz, Graz, Austria; ^3^Department of Education Research and Teacher Education, University of Graz, Graz, Austria

**Keywords:** ECG, psychophysiological data, educational psychology research, learning processes, motivation, objective/subjective data

## Introduction

Educational psychology has always been focused on improving learning and teaching practices (Münsterberg, 1914) by considering teachers' and learners' experiences, from children to adults. Its thematic focus has shifted over time to capture the growing complexity in learning and instruction. As a result, the range of methods has expanded: in addition to observations, (semi-)projective tests and questionnaires (McClelland et al., 1953; Schunk et al., 2007), psychophysiological measurements (Spangler et al., 2002) are also used in educational psychology to investigate performance and associated processes. This paper focuses on the latter with special consideration of the electrocardiogram (ECG), which provides valuable insights into learning processes.

## Added value of psychophysiological measurements

Knowledge of psychophysiology helps us understand processes crucial for learning, which underly psychological constructs such as drive, motivation, attitude, and emotion, and how they influence learning (Ax, 1964). Objectively measured psychophysiological data supports identifying risk factors, individual differences, and changes (over time). Moreover, they enable a more comprehensive understanding of individual responses and ultimately support the validity of the research results. Integrating objective/psychophysiological and subjective/self-reported data allows us to grasp the functional and practical dimensions of human behavior and underlying mental processes.

Non-invasive methods, e.g., functional magnetic resonance (fMRI; Murayama et al., 2010), or electroencephalography (EEG; Papousek et al., 2019), provide information on neural functionality and cortical activity. Methods like ECG (Spangler, 1997), skin conductance response (SCR; Järvenoja et al., 2020), or salivary cortisol (Kärner et al., 2017; Spangler et al., 2002), capture arousal and activation more easily. All these measurements record processes individuals may not be able to report on (Lackner et al., 2015; Wimmer et al., 2019), and cannot be distorted by social desirability (Pekrun, 2023). ECG offers significant advantages concerning high temporal resolution and precision, enabling real-time and highly accurate examination of heart function (Lackner et al., 2014; Vetta et al., 2023). This versatility underlines its importance for interdisciplinary studies, the complex interactions between cardiovascular physiology, autonomic functions (Kreibig, 2010), and emotions, which is crucial for understanding the links between physiology and psychology in learning.

## Autonomic nervous system functions

The autonomic nervous system (ANS) regulates vital basic functions such as neuroendocrine processes, organ function, adaptive responses to stress, or cardiovascular dynamics (Benarroch, 1993; Park and Thayer, 2014). The latter also concerns heart rate (HR) as well as heart rate variability (HRV). Whereas HR is quantified in beats per minute (bpm), and demonstrates rapid responsiveness to physiological and cognitive stimuli, HRV is indicative of the heart's capacity to adapt to environmental demands, reflecting alterations in the beat-to-beat rhythm (Berntson et al., 1997). The sympathetic nervous system (SNS) governs arousal states, mobilizing resources in response to stressors, while the parasympathetic branch (PNS) promotes restorative functions during calm periods (Lovallo and Sollers, 2000). Both systems modulate HR and HRV, with sympathetic dominance increasing cardiac activity and parasympathetic dominance decreasing it.

Concerning the analysis, HR is a recognized measure of arousal linked to mental demands, while HRV analysis offers various parameters, including time-domain and frequency-domain variables, reflecting different responses to stimuli or ANS balance (Task Force of the European Society of Cardiology the North American Society of Pacing Electrophysiology, 1996). Different coping processes, such as active coping associated with SNS dominance, and passive coping linked to heightened PNS activity, can be observed through increased HR or the heart's influence on blood pressure (Obrist et al., 1978). Moreover, cardiovascular reactivity reflects stress-induced changes, indicating adaptive resource use (McEwen, 1998; Richter et al., 2008), while recovery shows adaptability through baseline and post-stressor differences (Linden et al., 1997).

## The role of ECG in research in educational psychology

The examination of HR or cardiovascular arousal has revealed its association with the allocation of resources for the preparation and execution of actions (McEwen, 1998; Richter et al., 2008). Elevated HR during cognitive tasks indicates increased cerebral blood flow and activation of brain systems that aid in maintaining focus (Carroll et al., 2012; Duschek et al., 2010). A stronger HR response to cognitive demands is linked to greater effort and better task performance. Studies on students' cardiovascular responses found increased HR during oral presentations, with higher intensity observed when presenting to an audience compared to a laboratory setting (Kamarck et al., 2000; Turner et al., 1990). Moreover, research demonstrated higher HR reactivity during exams, with students reporting elevated state anxiety before real performance situations. Additionally, less resilient students exhibited no vagal recovery following an oral exam (Spangler, 1997). Another study found that lower vagal tone at the beginning of the semester predicted higher test anxiety at its end (Grabo et al., 2025), reinforcing the link between autonomic regulation and academic stress.

Further results demonstrate that motivation and (academic) emotions significantly influence students' cognition in typical school situations and regular classroom engagement: Mastromatteo et al. (2022), observed that students who were better able to regulate their psychophysiological responses (i.e., vagal tone) to stressful tasks, also showed higher participation in classroom activities. Thus, an improvement in coping with challenging tasks may also have beneficial effects on active participation in class. Moreover, interactive teaching methods in online learning environments have been shown to increase psychophysiological arousal, reflecting higher levels of engagement and cognitive activation (Gellisch et al., 2023). This underscores the importance of incorporating active learning elements into digital education, especially as distance learning becomes more prevalent. For good educational outcomes and student wellbeing, it is important to maintain student motivation by encouraging both cognitive challenge and active participation through sufficiently challenging tasks (Minkley et al., 2017). Cardiovascular recovery studies further underline the importance of addressing cognitive and emotional links between delayed recovery and ruminative thinking (Radstaak et al., 2011), providing insights into (mal)adaptive coping strategies in students. These findings emphasize the need to teach students effective stress management techniques such as mindfulness or cognitive reframing to reduce rumination and promote recovery from academic challenges. At the same time, they provide information on how to design learning environments that can teach and promote active engagement, healthy self-regulation and self-concept as well as adaptive coping strategies.

Overall, this body of research sheds light on how (learning) stimuli can evoke cognitive patterns reflected in cardiac responses, enhancing the value of ECG data alongside self-reported information. Additionally, the classroom, as a multi-actor system, emphasizes the crucial role of teachers in students' emotions too (Mainhard et al., 2022). Wellbeing in the classroom also includes teachers' psychophysiological data, as their HR reactivity reflects professional commitment. The integration of objective and subjective data contributes to understanding the perspectives of learners and teachers in typical or challenging educational situations, thereby expanding our exploration of learning processes.

Moreover, many conclusions and critical insights into students' stress levels and engagement may not be as clearly highlighted without psychophysiological measures. ECG measurements are widely used in research due to the availability of user-friendly systems and high-precision portable devices (Chan et al., 2022; Vetta et al., 2023). Their use is also beneficial in educational contexts such as classrooms (Kanna et al., 2018) and at university (Wimmer et al., 2019), as they are easy to use, have few limitations and enable real-time monitoring of psychophysiological processes. For use in schools, initial structures need to be established, as ECG protocols necessitate age- and gender-specific reference values, standardized data collection methods, and as far as possible automated analysis. But even more important than comprehensive implementation of ECG measures is that they are available when needed and, that the findings from representative studies are incorporated into teacher training and pedagogical concepts. So far, most research has focused on university students. However, issues such as exam anxiety could be counteracted earlier if there were more studies with large school student samples. Thus, on one hand, schools should be better involved in research and on the other hand, single measurements at schools make especially sense on a needs-oriented basis if targeted (individual) interventions can follow clarification.

### Remaining limitations and challenges

Despite the insights gained from ECG data in research, their integration entails increased effort, particularly concerning the investigation, analysis and interpretation, and implementation.

ECG data collection may lead to increased costs for researchers and participants. This includes financial expenses (Pekrun, 2023) for staff and equipment, compensation for participants, and additional time required for data collection and providing study information to each participant.

Analyzing ECG data requires an understanding of the electrophysiology of the heart and the principles of signal processing to correctly interpret the influence of the ANS. Selecting relevant parameters for analysis is crucial to address the research question effectively. Factors such as age, gender (Bonnemeier et al., 2003), the cognitive demand of the stimulus, the stimulus's ability to reflect cardiovascular alterations (Lackner et al., 2014), and the absence of a baseline reference (e.g., Mainhard et al., 2022; Turner et al., 1990) influence ECG characteristics and require careful consideration during technical analysis. Adjustments in HRV conventions and frequency bands may be needed based on sample characteristics (Lackner et al., 2020). Although automated ECG analyses are already quite advanced and efficient, certain expertise is still needed for specific adaptations. Interpreting HRV frequency-domain variables necessitates a deeper understanding of physiological and mathematical principles, with recommended adjustments based on sample age. Thereby, adjusting time resolutions according to expected effects can prevent confounding factors, such as HR mean values obscuring short-term effects in cognitively demanding paradigms (Lackner et al., 2014).

Successful ECG research or implementation in educational context depend—apart from the legal and financial requirements—on acceptance factors as well as on the direct use/knowledge gained of the results. First, awareness concerning the responsibility of educational institutions must be created, then responsibilities need to be clarified and finally training for the people involved is necessary (Cohen et al., 2017). The process of understanding and acceptance requires corresponding structures, time, and intensive communication of the benefits. Especially in schools with limited resources, several challenges may arise concerning study feasibility, cultural and ethical considerations. One major limitation is financial constraints when ECG devices are not funded. The cost of medical-grade ECG machines and consumables, such as electrodes and gels, can further restrict large-scale studies (e.g., Maurizi et al., 2023). Time constraints can complicate participation, as both students and teachers often have demanding schedules (Mukherjee, 2019). Finally, time and financial resources become a problem if the extra work for teachers cannot be compensated.

### Ethical considerations

Interpreting and classifying psychophysiological measures require additional information (Friedman et al., 2014), such as on the specific task (e.g., mental stress), the body position (e.g., sitting), age, gender, and health status (e.g., chronic illnesses, regular medication). Participants need to provide informed consent concerning the data collected, and experimenters-in-charge need to handle them responsibly because a data breach could result in the misuse of person related sensitive, as well as heath data. While there are well-established rules of good scientific practice for researchers, who are generally well trained, all of these have yet to be developed for educational context.

In addition to ethical considerations, cultural challenges also arise as ECG measurements involve direct skin contact which may cause skepticism or discomfort (Cohen et al., 2017). Given the potential inconvenience that ECG measurements may cause for participants, the effort for everyone involved, and the fact that a lot of personal data is collected, recordings must be conducted in a well-considered manner, ensuring participant protection, confidentiality, and follow-up medical care if needed.

## Conclusion and future perspectives

This opinion paper aims to show how ECG data can enhance educational psychology research by providing insights into psychophysiological processes involved in learning. While self-assessment questionnaires can allocate data to specific personality traits, ECG recordings offer additional information about perceptions and cognitions in the learning context in relation to individual characteristics. The comprehensive understanding that ECG data provide, makes its use in educational psychology highly valuable. We therefore agree with Pekrun (2023) that establishing a consistent knowledge base on psychophysiological data is essential and requires further research to develop uniform standards.

Finally, efforts should not only aim to increase validity, but also to ensure that the findings are used to improve learning processes and teaching practices and are consistent with the principles of educational psychology.
